# Effects of siRNA targeting BMPR-II on the biological activities of human liver cancer cells and its mechanism

**DOI:** 10.1186/1475-2867-14-55

**Published:** 2014-06-19

**Authors:** Peng Zeng, Sheng Cai, Jia-na Zhang, Feng-ming Yi, Wei-min Jiang, Jian-bing Wu

**Affiliations:** 1 Department of Oncology, the Second Affiliated Hospital, Nanchang University, Nanchang 330006, China; 2 Department of Pathology, the Second Affiliated Hospital, Nanchang University, Nanchang 330006, China; 3 Key Laboratory of Molecular Medicine Jiangxi Province, Nanchang 330006, China; 4 One number, Minde Road, Nanchang City, Jiangxi Province, China

**Keywords:** Liver cancer, Bone morphogenetic protein receptor II, Small interfering RNA, Mitogen-activated protein kinases, Vascular endothelial growth factor-C

## Abstract

**Background:**

Bone morphogenetic protein receptor II (BMPR-II) plays an important role in tumor’s invasion and proliferation. In this study, we observed the effects of small interfering RNA (siRNA) targeting bone morphogenetic protein receptor II (BMPR-II) on the biological activities of human liver cells and explore its mechanism.

**Methods:**

The molecular sequences of three siRNA targeting BMPR-IIwere designed and synthesized. In this study, there were 6 groups including group I (normal control), group II (blank control), group III (negative control) and group IV-VI (BMPR-II-siRNA-a, siRNA-b and siRNA-c-transfected cells, respectively). The levels of mRNA and protein of BMPR-II were determined to select the best sequence for BMPR-II silence. After liver cancer cells were transfected with the best sequence, proliferation and invasion of transfected cells were assessed, and apoptosis and cell cycle were detected. The expressions of mitogen-activated protein kinases (MAPKs) signal pathway-related VEGF-C protein were observed after BMPR-II silence and BMPR-II silence combined with inhibiting MAPKs signal pathway, respectively.

**Results:**

RT-PCR and Western blot indicated that BMPR-II expression was the highest in HepG2 among the three liver cancer lines (*P* < 0.01) and the lowest in group IV among the six groups (*P* < 0.01). MTT assay and transwell assay revealed that the numbers of cell growth and cell transmembrane were significantly lower in group IV than in control groups 48 h after cells were transfected (*P* < 0.05). Flow cytometer showed that apoptosis was the highest and cells were significantly blocked in S phase 48 h after cells were transfected in group IV (*P* < 0.01). Western blot indicated that the protein levels of p-P38 (*P* < 0.01) and vascular endothelial growth factor-C (VEGF-C) (*P* < 0.01) were significantly decreased after BMPR-II silence. The protein level of VEGF-C was significantly decreased in PD98059 + siRNA-BMPR-II-a and SB203580 + siRNA-BMPR-II-a groups (*P* < 0.01), especially in SB203580 + siRNA-BMPR-II-a group (*P* < 0.01).

**Conclusions:**

siRNA targeting BMPR-IIcan markedly inhibit HepG2 proliferation and invasion, promote apoptosis and block HepG2 in S phase. Its mechanism may be that BMPR-II silence down-regulates VEGF-C expression through MAPK/P38 and MAPK/ERK1/2 pathways, especially MAPK/P38. This study provides a new targeted therapy for liver cancer.

## Introduction

Bone morphogenetic proteins (BMPs), a member of transforming growth factor beta (TGF-β) family [1], are involved in cell proliferation, migration, differentiation and apoptosis [2]. Neovascularization is important for tumor’s invasion and metastasis. Vascular endothelial growth factor (VEGF) plays an important role in solid tumor’s growth, progression, metastasis, proliferation and differentiation [3]. VEGF-C is present not only in endothelial cells, but also in tumor cells, and plays regulatory roles in tumor angiogenesis and lymphogenesis [4]. BMPs also play an important role in embryonic angiogenesis [5]. However, BMPs perform their biological functions through its receptor, bone morphogenetic protein receptor II (BMPR-II). BMPR-II plays an important role in tumor’s invasion and proliferation [6,7]. Many physiologic functions of BMP-II are achieved through activating mitogen-activated protein kinase (MAPK) and PI3K pathways [8,9]. MAPK is an important signal transduction system in cells and a converging point of various signal pathways. ERK1/2 pathway is mainly involved in cell growth and differentiation, while JNK and p38 pathways participate in stress reactions such as inflammation and apoptosis [10]. Little research has been done about the effects of BMPR-II on invasion and proliferation of human liver cancer cells and its mechanism. Therefore, we observed the effects of small interfering RNA (siRNA) targeting BMPR-II on the invasion, proliferation, apoptosis and cell cycle of liver cancer cells and explored its mechanism. This study provides a theoretical and experimental basis for exploring the occurrence and progression of human liver cancer.

## Results

### Screening the cell line with higher expression of BMPR-II from liver cancer cell lines HepG2, SMMC7721 and Hep3B

**Figure 1:**
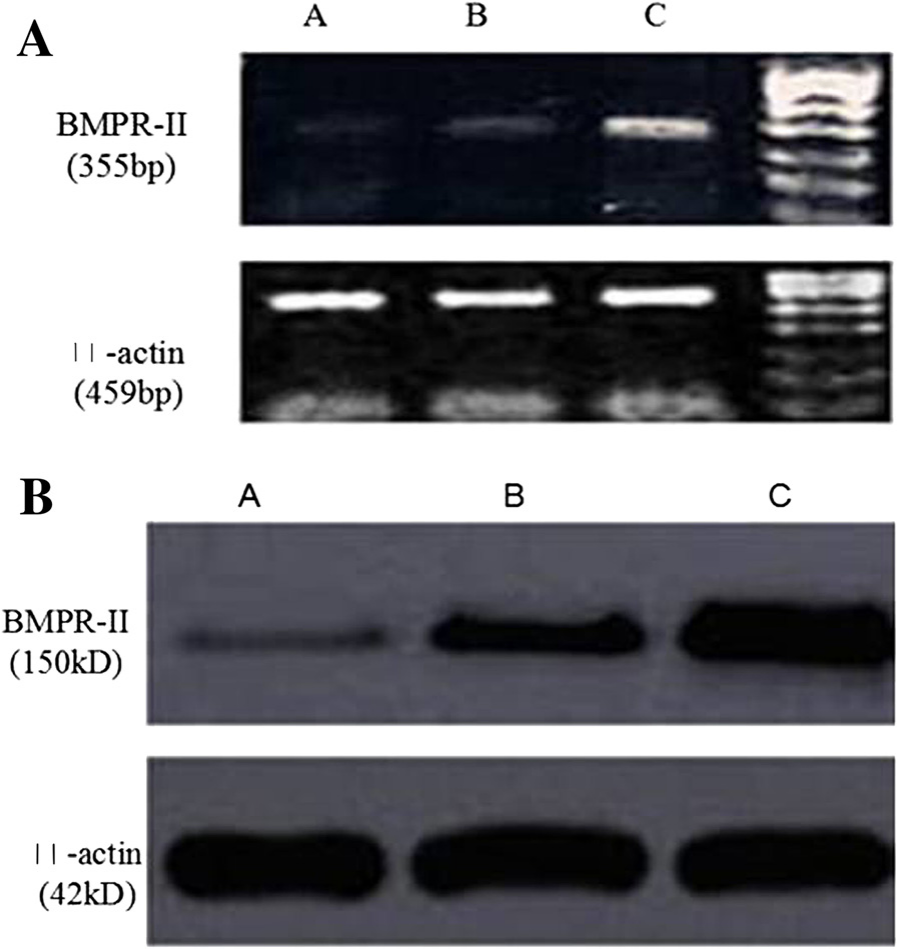
**Expressions of BMPR-II mRNA and protein in each cell line. A**: Expressions of BMPR-II mRNA in each cell line. **B**: Expressions of BMPR-II protein in each cell line. A: Hep3B cells; B: SMMC7721 cells; C: HepG2 cells.

RT-PCR showed that the ratios of BMPR-II to β-actin in Hep3B, SMMC7721 and HepG2 were 0.58 ± 0.00, 0.76 ± 0.05 and 1.00 ± 0.04, respectively, and Western blot showed that the ratio of BMPR-II to β-actin were 0.48 ± 0.07, 0.65 ± 0.44 and 1.01 ± 0.06, respectively. Therefore, the expression of BMPR-II in HepG2 cells was the highest among the three liver cancer cell lines (*P* < 0.01) (Figure 1).

### Transfection efficiency

Green fluorescence could be seen under a fluorescence microscope when cells had been successfully transfected with siRNA, because siRNAs carried fluorescence mark. The siRNA transfection efficiency was the highest at 80% when cells were transfected with 50 nmol/L of siRNA for 12 h.

### Expressions of BMPR-II mRNA and protein after BMPR-II silence in HepG2 cells

**Figure 2:**
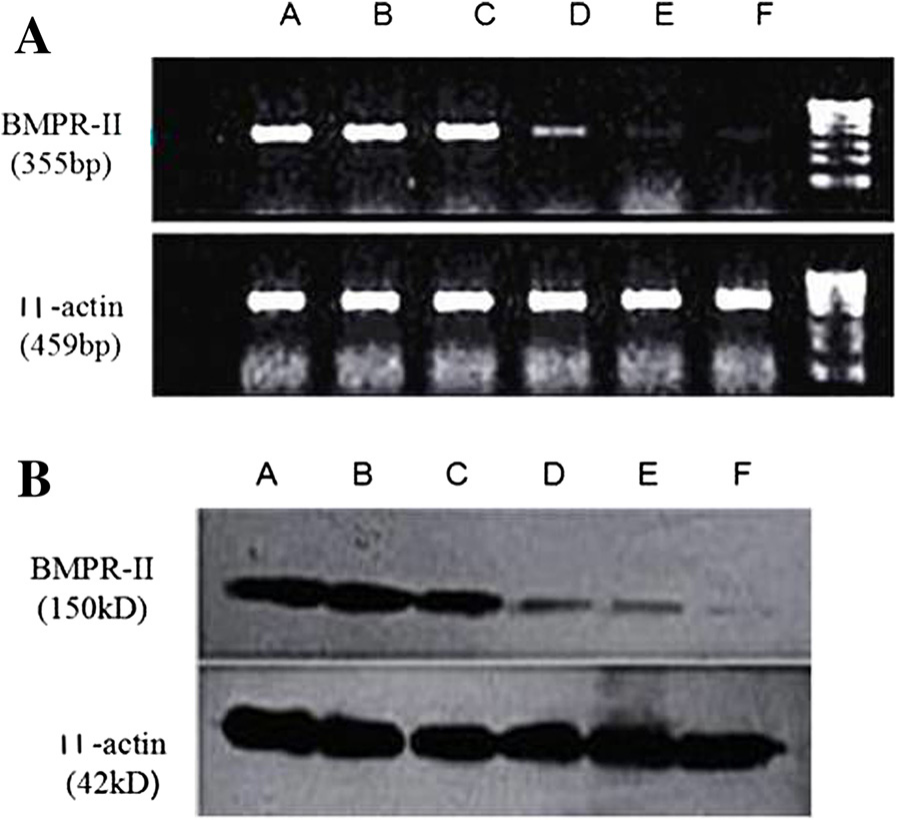
**Expressions of BMPR-II mRNA and protein in each group. A**: Expressions of BMPR-II mRNA in each group. **B**: Expressions of BMPR-II protein in each group. A: Normal group; B: Blank control group; C: Negative control group; D: BMPR-II-siRNA-c group; E: BMPR-II-siRNA-b group; F: BMPR-II-siRNA-a group.

RT-PCR revealed that the absorbance ratios of groupI-VI were 0.9 ± 0.07, 0.89 ± 0.10, 0.90 ± 0.10, 0.20 ± 0.01, 0.36 ± 0.04 and 0.56 ± 0.02, respectively. Western blot indicated that the gray scale ratios of groupI-VI were 0.95 ± 0.03, 0.98 ± 0.03, 0.88 ± 0.02, 0.39 ± 0.02, 0.53 ± 0.01 and 0.60 ± 0.01, respectively. The expressions of BMPR-II mRNA and protein were significantly lower in the three specific transfection groups (group IV-VI) than other groups (*P* < 0.01), especially in group IV (*P* < 0.01, Figure 2).

### Effects of BMPR-II silence on the growth and morphology of HepG2 cells

**Figure 3:**
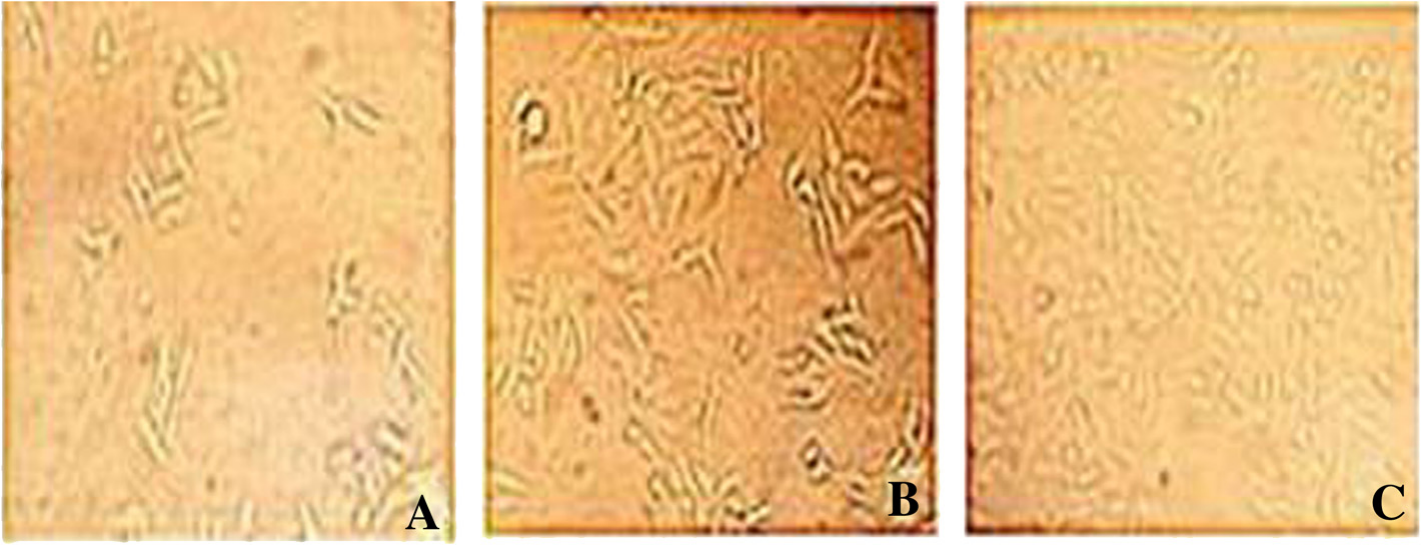
**Effects of BMPR-II silence on the growth and morphology of HepG2 cells (×200). A**: BMPR-II-siRNA-a group; **B**: Negative control group; **C**: Normal control group.

Cell growth and morphology were observed under an invert microscope 48 h after cells were transfected with siRNA targeting BMPR-II. Cells grew well with good refractivity in normal control and negative control groups. In siRNA-BMPR-II-a group, cells were shrunken with poor refractivity and cell debris, and adherent cells were significantly reduced (Figure 3).

### MTT assay

MTT assay indicated that there were no statistical differences in survival rate of HepG2 cells between the three groups when cells were transfected for 24 h (*P* > 0.05), but the survival rate of HepG2 cells were lower in BMPR-II-siRNA-a group (48.27% ± 0.76% and 46.03% ± 0.62%) than in negative control group (81.21% ± 0.80% and 79.18% ± 0.68%) and normal control group (82.64% ± 0.67% and 81.55% ± 0.71%) when cells were transfected for 48 h and 72 h, respectively (*P* < 0.05). There was also no statistical difference in survival rate between HepG2 cells treated for 48 h and 72 h in BMPR-II-siRNA-a group.

### Transwell assay

**Figure 4:**
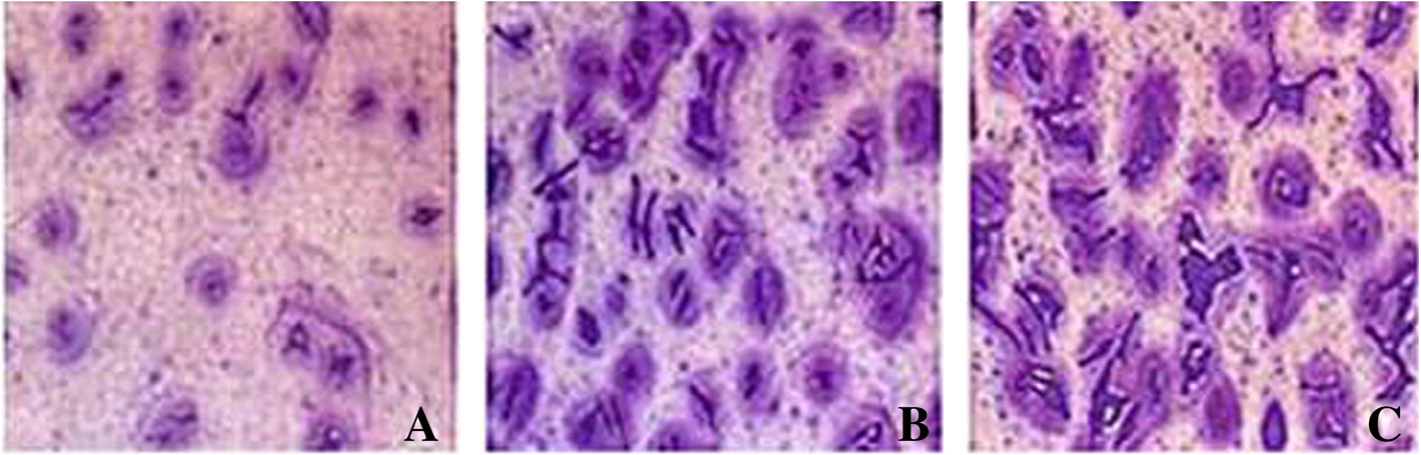
**Effects of BMPR-II silence on the invasion of HepG2 cells (×200). A**: BMPR-II-siRNA-a group; **B**: Negative control group; **C**: Normal control group.

The number of cells to penetrate matrigel from Transwell upper chamber to Transwell lower chamber reflects the ability of cell invasion. Five visual fields in each group were randomly selected to count the number of invading cells. The number of cells to penetrate the membrane was significantly lower in BMPR-II-siRNA-a group (25.20 ± 1.60) than in negative control group (59.50 ± 1.85) and normal control group (60.40 ± 1.39) (*P* < 0.05). The results indicated that the invasion ability of liver cancer HepG2 cells was significantly decreased after the cells were treated with BMPR-II-siRNA-a (Figure 4).

### Effects of BMPR-II silence on liver cancer HepG2 apoptosis

**Figure 5:**
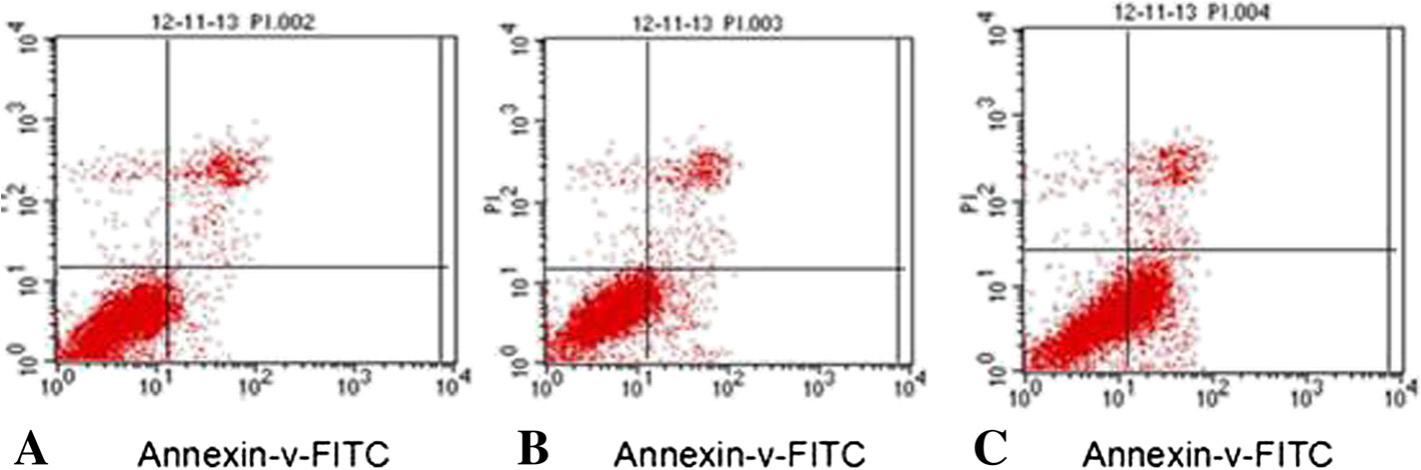
**Effects of BMPR-II silence on liver cancer HepG2 apoptosis. A**: Normal control group; **B**: Negative control group; **C**: BMPR-II-siRNA-a group.

Flow cytometer indicated that apoptosis was significantly higher in BMPR-II-siRNA-a group (37.0 ± 30.56, transfected for 48 h) % than in normal control group (5.36 ± 1.34) % and negative control group (9.53 ± 0.23) % (*P* < 0.01, Figure 5).

### Effects of BMPR-II silence on cell cycle of liver cancer HepG2

**Figure 6:**
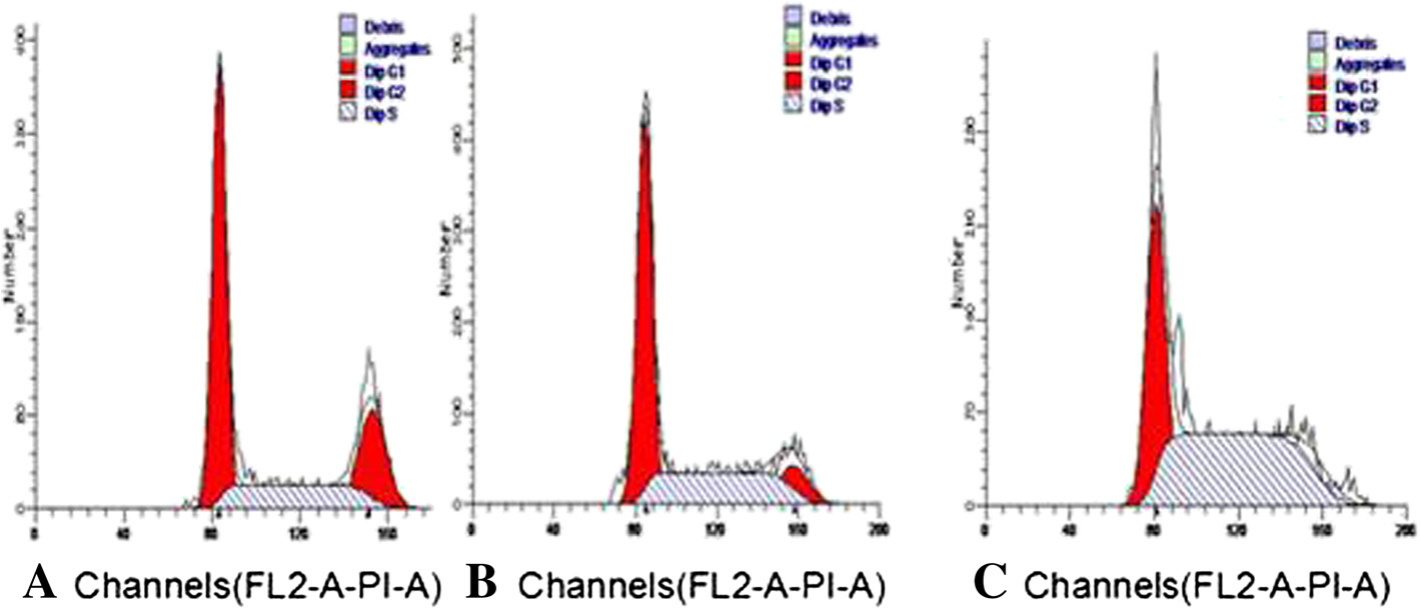
**Effects of BMPR-II silence on cell cycle of liver cancer HepG2. A**: Normal control group; **B**: Negative control group; **C**: BMPR-II-siRNA-a group.

Flow cytometer indicated that cells were significantly blocked in S phase 48 h after cells were transfected in BMPR-II-siRNA-a group; but in normal control and negative control groups, cell cycle was not markedly changed (Table 1, Figure 6).

**Table 1:** Effects of BMPR-II silence on cell cycle in three groups (x ± s, n = 3)

**Group**	**G0/G1**	**S**	**G2/M**
Normal control group	46.83 ± 5.76	30.21 ± 5.50	22.96 ± 0.94
Negative control group	47.50 ± 5.76	39.50 ± 0.89	14.68 ± 3.50
BMPR-II-siRNA-a group	38.02 ± 2.06	50.63 ± 13.09*	5.35 ± 1.26

*P < 0.01 vs G0/G1 and G2/M.

### Effects of BMPR-II silence on the expressions of MAPK signal pathway-related proteins and VEGF-C protein

Western blot indicated that the protein expressions of BMPR-II, VEGF-C, p-P38 and p-ERK1/2 were significantly lower in BMPR-II-siRNA-a group than in normal control and negative control groups (*P* < 0.01, Table 2), but there were no significant differences in p-JNK protein expression between the three groups (*P* > 0.05, Table 2).

**Table 2:** Expressions of MAPK signal pathway-related proteins and VEGF-C protein after BMPR-II silence in HepG2 cells (RGS, x ± s, n = 3)

**Group**	**A**	**B**	**C**
BMPR-II	0.95 ± 0.02	0.92 ± 0.04	0.46 ± 0.02*
VEGF-C	0.97 ± 0.03	0.82 ± 0.03	0.33 ± 0.05*
p-P38	0.95 ± 0.04	0.86 ± 0.06	0.45 ± 0.05*
p-ERK1/2	0.98 ± 0.05	0.90 ± 0.04	0.35 ± 0.03*
	0.97 ± 0.03	0.86 ± 0.03	0.32 ± 0.04*
p-JNK	0.93 ± 0.02	0.90 ± 0.03	0.87 ± 0.05
P38	0.97 ± 0.04	0.98 ± 0.05	0.96 ± 0.03
ERK1/2	0.90 ± 0.03	0.96 ± 0.05	0.97 ± 0.05
JNK	0.98 ± 0.05	0.94 ± 0.04	0.96 ± 0.03

Notes: BMPR-II: bone morphogenetic protein receptor II; RGS: relative gray scales; A: normal control group; B: negative control group; C: BMPR-II-siRNA-a group.

*P < 0.01 *vs* group A and B.

### Effects of specific inhibitors (SB203580, PD98059 and SP600125) on the expression of VEGF-C protein

Western blot revealed that the gray scale ratios of VEGF-C were lower in PD98059 group (0.55 ± 0.03) and SB203580 group (0.41 ± 0.03) than in SP600125 group (0.94 ± 0.03) and normal control group (0.98 ± 0.01) (P < 0.01), and in SB203580 group (0.41 ± 0.03) than in PD98059 group (0.55 ± 0.03) (P < 0.01).

### Expressions of MAPK signal pathway-related proteins and VEGF-C protein after BMPR-II silence combined with inhibiting MAPK signal pathway

The expressions of MARK signal pathway-related proteins and VEGF-C protein after BMPR-II silence combined with inhibiting MAPK signal pathway are shown in Table 3. Table 3 showed that the corresponding protein expression was down-regulated after each signal pathway was blocked. However, the gray scale ratios of VEGF-C were lower in BMPR-II-siRNA-a + PD98059 group (0.44 ± 0.02 ) and BMPR-II-siRNA-a + SB203580 group (0.34 ± 0.02) than in BMPR-II-siRNA-a + SP600125 group (0.79 ± 0.01) and normal control group (0.79 ± 0.02) (P < 0.01), and in BMPR-II-siRNA-a + SB203580 group (0.34 ± 0.02) than in BMPR-II-siRNA-a + PD98059 group (0.44 ± 0.02) (P < 0.01).

**Table 3:** Expressions of MAPK signal pathway-related proteins and VEGF-C protein after BMPR-II silence combined with inhibiting MAPK signal pathway in HepG2 cells (RGS, x ± s, n = 3)

** Group**	**A**	**B**	**C**	**D**
p-ERK1/2	0.20 ± 0.04	0.87 ± 0.03	0.88 ± 0.05	0.82 ± 0.03
	0.36 ± 0.03	0.90 ± 0.03	0.92 ± 0.05	0.91 ± 0.06
p-P38	0.85 ± 0.04	0.34 ± 0.05	0.84 ± 0.05	0.89 ± 0.02
p-JNK	0.87 ± 0.04	0.91 ± 0.02	048 ± 0.02	0.91 ± 0.01
VEGF-C	0.44 ± 0.02 *	0.34 ± 0.02*	0.79 ± 0.01	0.79 ± 0.02

Notes: BMPR-II: bone morphogenetic protein receptor II; RGS: relative gray scales; A: BMPR-II-siRNA-a + PD98059 group; B: BMPR-II-siRNA-a + SB203580 group; C: BMPR-II-siRNA-a + SP600125 group; D: normal control group.

*P < 0.01 *vs* group C and D. #*P* < 0.01 *vs* group A.

## Discussion

BMPs, a group of functional proteins, are widely involved in proliferation, differentiation and apoptosis of many cells, and play an important role in tumor’s proliferation, invasion and metastasis. BMPs perform their biological functions through their receptors, BMPR-II. BMPR-II gene mutation allows BMPR-II-mediated BMP signal transduction to inactivate, leading to carcinogenesis [11]. Park et al. [11] have reported that the activation of BMPR-II–mediated BMP signal pathway is one of mechanisms of stomach or colon cancer development. Ye et al. [12] have described that hepatocyte growth factor can up-regulate BMPR-IB; and in prostate cancer, BMPR-II promotes bone metastasis of prostate cancer.

Angiogenesis is strongly associated with tumor’s growth, invasion and metastasis [13], and VEGF-C plays an important role in angiogenesis and lymphangiogenesis [14].

MAPKs, a kind of serine/threonine kinase, are an important signal transduction system in cells and a converging point of various signal pathways. ERK1/2 pathway is mainly involved in cell growth and differentiation. JNK and p38 pathways are called stress-activated protein kinase (SAPK) because they participate in stress reactions such as inflammation and apoptosis. JNK and p38 pathways are also involved in cell proliferation and differentiation, and respond to extracellular stimuli [15]. Surgical injury can cause inflammatory reaction to stimulate the production of P38MAPK, in the same, solid tumor also can release cytokine to stimulate the production of P38MAPK [16].

It is reported that Ras can up-regulate VEGF expression through activating Raf-mek-ERK1/2-MAPK pathway [17]. Compared with p-ERK, BMP receptor activation more readily mediates p-P38 activation, and BMP-2 is strongly associated with VEGF in angiogenesis [18]. However, the specific subtype and signal pathway of BMPR-II are not clear yet. Therefore, we observed the changes in liver cancer cell’s invasion, proliferation, apoptosis and cell cycle; and the changes in MAPK signal pathway-related proteins (p-P38, p-ERK1/2, p-JNK and VEGF-C) after BMPR-II silence to explore the biomechanism that BMPs affect liver cancer’s invasion, proliferation and metastasis.

In previous study, we found that BMP-2 could promote liver cancer cell’s proliferation and migration, and played an important role in liver cancer invasion through down-regulation of MMP2 and MMP9 [19]. However, BMPR-II mechanisms about vascular invasion and lymphatic metastasis are not clear in liver cancer. Therefore, in this study, BMPR-II gene was interfered with siRNA. We first selected the liver cancer HepG2 cells with the highest expression of BMPR-II from three liver cancer cell lines, then observed the changes in liver cancer cell’s invasion, proliferation, apoptosis and cell cycle after BMPR-II silence; and the changes in MAPK signal pathway-related proteins (p-P38, p-ERK1/2, p-JNK and VEGF-C) after BMPR-II silence, and BMPR-II silence combined with inhibiting MAPK signal pathway (with SB203580: P38 inhibitor, PD98059: ERK1/2 inhibitor and SP600125: JNK inhibitor, respectively). Our results indicated that BMPR-II expression was the highest in HepG2; after HepG2 was transfected with BMPR-II-siRNA-a, invasion and proliferation of HepG2 was significantly decreased, but HepG2 apoptosis was significantly increased, and HepG2 cells were significantly blocked in S phase. At the same time, we also found that after BMPR-II silence, MAPKs signal pathway-related proteins p-P38 and p-ERK were significantly down-regulated and VEGF-C protein was also down-regulated, but p-JNK was unchanged. Subsequently, after MAPKs-related pathways were inhibited, we found that P38 and ERK signal pathways were inhibited with down-regulation of VEGF-C expression, but JNK signal pathway was inhibited with unchanged VEGF-C expression. In order to explore the relationship between BMPR-II-ERK/P38 and VEGF-C, VEGF-C expression after BMPR-II silence combined with inhibiting MAPK sub-signal pathways was observed, results indicated that VEGF-C expression was significantly down-regulated in P38 group (si-BMPR-II + SB203580) and ERK group (si-BMPR-II + PD98059), especially in p-P38 group, but was unchanged in JNK group (si-BMPR-II + SP600125). Based on the results above, we conclude that for human liver cancer HepG2, specific siRNA targeting BMPR-IIcan markedly inhibit cell proliferation and invasion, promote cell apoptosis and block cells in S phase. Its mechanism may be that BMPR-II silence down-regulates VEGF-C expression through MAPK/P38 and MAPK/ERK1/2 pathways, especially MAPK/P38.

The clinical treatment for liver cancer is difficult due to its malignant biological characteristics such as invasion and metastasis. In this study, we explored the mechanisms of liver cancer’s proliferation, invasion and metastasis, providing a new targeted therapy for liver cancer.

BMPs promote VEGF expression, and VEGF finally affects vascular endothelial cells. Whether BMPs directly promote vascular endothelial cell proliferation will be investigated in our further studies.

## Materials and methods

### Reagents

Human liver cancer cell lines (HepG2, SMMC7721 and Hep3B) were provided by Jiangxi Province Key laboratory of molecular medicine (Nanchang, China). Fetal calf serum DMEM, RPMI1640 and MEM medium were purchased from Hyclone (Logan, USA). Trypsin was from Solarbio (Beijing, China). Trizol reagent was provided by Tiangen (Beijing, China). RT kit was purchased from TAKALA (Kyoto, Japan). The primers of BMPR-II and control β-actin were synthesized by Invitrogen (Carlsbad, USA). Annexin V-EGFP Apoptosis Detection Kit was purchased from Multiscience (Hangzhou, China). Cell cycle kit was purchased from KEY GEN (Nanjing, China). Lipofectamine 2000 was purchased from Invitrogen (Carlsbad, USA).Mouse anti-human monoclonal antibodies of p-JNK and β-actin; and rabbit anti-human polyclonal antibodies of BMPR-II, p-ERK1/2, p-p38 and VEGF-C were purchased from CST (Boston, USA). Secondary antibodies of goat anti-mouse and goat anti-rabbit were purchased from Beijing Zhongshan Golden Bridge Bio (Beijing, China).

### Cell recovery and culture

Liver cancer HepG2, SMMC7721 and Hep3B cells were taken from liquid nitrogen, and then thawed in 37°C water bath followed by quickly placing in DMEM medium containing 10% fetal calf serum at 37°C in an atmosphere of 5% CO2 until they grew covering 90% of the culture bottle. These cells were seeded in new culture bottles after they were digested with 0.25% trypsin.

### RT-PCR to select the cell line with highest level of BMPR-II mRNA from HepG- 2, SMMC7721 and Hep3B cell lines

Total RNA was extracted from cells using one-step method. The primers of BMPR-II and control β-actin were synthesized by Invitrogen (Carlsbad, USA). The upstream of BMPR-II primer was 5′-CAAGAACGGCTATGTGCG-3′ and downstream 5′-CTCGGTTAAATGAATGAGG- TG-3′ with a length of 355 bp of PCR product. The upstream of control β-actin primer was 5′-TAAGAAGCTGCTGTGCTACG-3′ and downstream, 5′-GACTCGTCATACTCCTGCTT- 3′with a length of 459 bp of PCR product. PCR conditions were as follows: pre-denaturing at 94°C for 5 min, denaturing at 94°C for 40 s, reannealing at 63°C for 30 s, elongation at 72°C for 40 s, 30 cycles; finally elongation at 72°C for 8 min. PCR products underwent 1% agarose gel electrophoresis.

### Western blot to select the cell line with highest level of BMPR-II protein from HepG- 2, SMMC7721 and Hep3B cell lines

Pre-cooled 4°C lysis buffer (volume of five times) was added in differently cultured cells to extract protein. The protein was stored at -20°C for future use. Protein concentration was determined using BCA method. Protein and buffer were mixed at a ratio of 5:1, and then placed in boiling water for 5–10 min.

A total of 80 μg of sample per well underwent 12% SDS-PAGE, and then was transferred onto nitrocellulose membrane followed by sealing using 10 ml of TBST containing 0.5% dried skim milk at 4°C overnight. Following washing two times using TBST with each time for several seconds, rabbit anti-human antibody of BMPR-II (1:1000) was added at 4°C overnight. Next day, following washing three times using TBST with each time for 10 min, HRP-labeled goat anti-rabbit and goat anti-mouse IgG (1:5000) were added for 2 h. The mixture was washed three times using TBST with each time for 10 min followed by visualization using substrate electrochemiluminescence.

### Design and synthesis of sequences of siRNAs targeting BMPR-II

The three pairs of sequences of siRNAs targeting BMPR-II were designed according to hBMPR-II mRNA (NM_001200) published by Genbank. These siRNAs were called BMPR-II-siRNA-a, BMPR-II-siRNA-b and BMPR-II-siRNA-c, and a negative control sequences was also designed. The transcription templates of siRNAs were synthesized by Invitrogen (Carlsbad, USA) and their sequences are shown in Table 4.

**Table 4:** Sequences of Specific BMPR-II-siRNAs and Negative siRNA Targeting BMPR-II

**Name**	** Sequence (5′–3′)**
siRNA-BMPR-II-a	Positive-sense strand:5′-GCAAUUUCCCAUCGAGAUUTT-3′
	Anti-sense strand:5′-AAUCUCGAUGGGAAAUUGCTT-3′
siRNA-BMPR-II-b	Positive-sense strand:5′-CCAAGAGACCUACUAGUUUTT-3′
	Anti-sense strand:5′-AAACUAGUAGGUCUCUUGGTT-3′
siRNA-BMPR-II-c	Positive-sense strand:5′-GCAGCAAGCACAAAUCAAATT-3′
	Anti-sense strand:5′-UUUGAUUUGUGCUUGCUGCTT-3′
Nagative siRNA	Positive-sense strand: 5′-CAACCUUGCGGCCUUAGGGTT-3′
	Anti-sense strand 5^′^-UUGGCCCAAUUUCCCGGGCTT-3′

Notes: BMP: bone morphogenetic protein; BMPR-II: bone morphogenetic protein receptor-II; siRNA: small interfering RNA.

### Grouping and transfection

In this study, there were 6 groups including group I (normal control, non-transfected cells), group II (blank control, only liposome-transfected cells), group III (negative control, non-specific siRNA-transfected cells) and group IV-VI (BMPR-II-siRNA-a, siRNA-b and siRNA-c-transfected cells, respectively). Cells were adjusted to 1 × 10^5^/ml, and then seeded in 6-well plate for 24 h. Cells covering 70–80% of the hole wall were incubated in 3 ml of serum-free DMEM for 2 h followed by transfection according to the instructions of kit. Five hours later, cells were incubated in DMEM containing 10% fetal calf serum for 24 h or 48 h followed by extraction of RNA or protein.

### Western blot and RT-PCR to select the best sequence of siRNA targeting BMPR-II from the three sequences

The cells transfected for 24 h or 48 h were used for extraction of RNA or protein, and then the levels of mRNA and protein of were determined with RT-PCR and Western blot. RT-PCR and Western blot were performed as previously described.

### MTT assay to assess the proliferation of liver cancer cells

There were normal control group, negative group and BMPR-II-siRNA-a group. Cells were seeded in a 96-well plate at a density of 1 × 10^5^ cells/well with a final volume of 100 μl. After cells covering 70–80% of the hole wall were transfected for 24, 48 and 72 h, respectively; 20 μl of MTT was added at 37°C for 4 h. After the supernatant fluid was removed, 150 ml of DMSO was added for 10 min with shaking. Cell viability was assessed by measuring the absorbance at 490 nm using an Enzyme-labeling instrument (EX-800 type). All measurements were performed in triplicate. The results were expressed as the average of three independent experiments.

### Transwell assay to evaluate the invasion of liver cancer cells

Cell invasion was assessed using 8 μm pore size Borden chamber (24-well plate). Borden chamber was washed using serum-free DMEM, and then 50 μl of Matrigel (1:8) was used to coat the upper surface of the filter. After cells in each group (normal control group, negative control group and BMPR-II-siRNA-a group) were transfected for 48 h, 200 μl of cell suspension (1 × 10^5^/ml) was added in the upper compartment with serum-free medium containing 10 g/L of BSA, and then 500 μl of 10% fetal bovine serum was added in the lower compartment. Borden chamber was placed in an atmosphere of 5% CO2 at 37°C for 24 h followed by washing with PBS. The cells not to penetrate the membrane of the upper chamber were wiped out using cotton swabs. The cells to penetrate the membrane were fixed with 95% of ethanol for 5 min, and then stained with 4 g/L of crystal violet. The cell-invading numbers in five visual fields were counted under a light microscope and the mean was calculated. The invasive ability of tumor cells was expressed as the cell-invading number. Testing was performed in triplicate in each group.

### Apoptosis and cell cycle to detect with flow cytometer

The samples were washed twice and adjusted to a concentration of 1 × 10^6^ cells/ml with 4°C PBS. The Falcon tubes (12 mm × 75 mm, polystyrene round bottom) were used in this experiment. For some samples, 100 μl of suspensions, 10 μl of annexin V-FITC and 10 μl of propidiom iodide (PI, 20 μg/ml) were respectively added into the labeled tube to detect apoptosis at room temperature in the dark for 10 min. Other samples were fixed with 70% of ice ethanol at 4°C for 12 h, and then treated with PI for 30 min to observe cell cycle.

### Effects of BMPR-II silence on the expressions of MAPK signal pathway-related proteins and VEGF-C protein

Total protein was extracted from the cells transfected with siRNA targeting BMPR-II for 48 h. The protein expressions of p-P38/P38, p-ERK1/2/ERK1/2, p-JNK/JNK and VEGF-C were detected with Western blot.

### Expression of VEGF-C protein after inhibiting MAPK signal pathway with SB203580, PD98059 and SP600125, respectively

Cells were adjusted to 1 × 10^5^/ml, seeded in 6-well plate, and then treated with serum-free DMEM for 2 h followed by addition of SB203580 (P38 inhibitor, 10 uM), PD98059 (ERK1/2 inhibitor, 40 uM) and SP600125(JNK inhibitor, 50 uM), respectively. Two hours later, protein was extracted, and then the level of VEGF-C protein in each group was determined with Western blot respectively.

### Expression of VEGF-C protein after BMPR-II silence combined with inhibiting MAPK signal pathway

Cells were adjusted to 1 × 10^5^/ml, seeded in 6-well plate, and then transfected with siRNA targeting BMPR-II for 48 hours. These transfected cells were treated with DMEM for 1–2 followed by addition 50 μm of SB203580 (P38 inhibitor, 10 uM), PD98059 (ERK1/2 inhibitor, 40 uM) and SP600125 (JNK inhibitor, 50 uM), respectively. Two hours later, protein was extracted, and then the expression of VEGF-C protein in each group was determined with Western blot.

### Statistical analysis

Statistical treatment was performed with SPSS 19.0 software. All data were expressed as x ± s (mean ± s.d.). Single-factor analysis of variance was used for comparison among multiple groups. *t*-test was used for comparison between two groups. Statistical significance was established at *P* < 0.05.

## Abbreviations

siRNA: Small interfering RNA; BMPR-II: Bone morphogenetic protein receptor II; MAPKs: Mitogen-activated protein kinases; BMPs: Bone morphogenetic proteins; TGF-β: Transforming growth factor beta; VEGF: Vascular endothelial growth factor; SAPK: Stress-activated protein kinase.

## Competing interests

There was no any competing financial interest in relation to the work.

## Author’s contributions

PZ: had made acquisition of data and involved in drafting the manuscript. SC: had made acquisition of data. JZ: had made acquisition of data. FY: had made analysis and interpretation of data. WJ: had made acquisition of data. JW: had made substantial contributions and given final approval of the version to be published. All authors read and approved the final manuscript.

## References

[B1] Maegdefrau U Bosserhoff AK BMP activated Smad signaling strongly promotes migration and invasion of hepatocellular carcinoma cells Exp Mol Pathol 2012 92 74 81 10.1016/j.yexmp.2011.10.004 22024355

[B2] Heinke J Kerber M Rahner S Mnich L Lassmann S Helbing T Heinke J Kerber M Rahner S Mnich L Lassmann S Helbing T Werner M Patterson C Bode C Moser M Bone morphogenetic protein modulator BMPER is highly expressed in malignant tumors and controls invasive cell behavior Oncogene 2012 31 2919 2930 10.1038/onc.2011.473 22020334 PMC3376172

[B3] Song G Li Y Zhang Z Ren X Li H Zhang W Song G Li Y Zhang Z Ren X Li H Zhang W Wei R Pan S Shi L Bi K Jiang G C-myc but not Hif-1α-dependent down-regulation of VEGF influences the proliferation and differentiation of HL-60 cells induced by ATRA Oncol Rep 2013 29 2378 2384 23588859 10.3892/or.2013.2395

[B4] Chen JC Chang YW Hong CC Yu YH Su JL The role of the VEGF-C/VEGFRs axis in tumor progression and therapy Int J Mol Sci 2012 14 88 107 10.3390/ijms14010088 23344023 PMC3565253

[B5] Pi X Schmitt CE Xie L Portbury AL Wu Y Lockyer P Pi X Schmitt CE Xie L Portbury AL Wu Y Lockyer P Dyer LA Moser M Bu G Flynn EJ 3rd Jin SW Patterson C LRP1-dependent endocytic mechanism governs the signaling output of the bmp system in endothelial cells and inangiogenesis Circ Res 2012 111 564 574 10.1161/CIRCRESAHA.112.274597 22777006 PMC3495066

[B6] de Carvalho CH Nonaka CF de Araújo CR de Souza LB Pinto LP Immunoexpression of bone morphogenetic protein -2(BMP-2), BMP receptor typeIA, and BMP receptor typeII in metastatic and non-metastatic lower lip squamous cellcarcinoma Oral Pathol Med 2011 40 181 186 10.1111/j.1600-0714.2010.00974.x 21059107

[B7] Herrera B van Dinther M Ten Dijke P Inman GJ Autocrine bone morphogenetic protein-9 signals throughactivin receptor-like kinase-2/Smad1/Smad4 to promoteovarian cancer cell proliferation Cancer Res 2009 69 9254 9262 10.1158/0008-5472.CAN-09-2912 19996292 PMC2892305

[B8] Viñals F López-Rovira T Rosa JL Ventura F Inhibition of PI3K/p70 S6K and p38 MAPK cascades increases osteoblastic differentiation induced by BMP-2 FEBS Lett 2002 510 99 104 10.1016/S0014-5793(01)03236-7 11755539

[B9] Guicheux J Lemonnier J Ghayor C Suzuki A Palmer G Caverzasio J Activation of p38 mitogen-activated protein kinase and c-Jun-NH2-terminal kinase by BMP-2 and their implicationin the stimulation of osteoblastic cell differentiation J Bone Miner Res 2003 18 2060 2068 10.1359/jbmr.2003.18.11.2060 14606520

[B10] Xu GJ Cai S Wu JB Effect of insulin-like growth factor-1 on bone morphogenetic protein-2 expression in hepatic carcinomaSMMC7721 cells through the p38 MAPK signaling pathway Asian Pac J Cancer Prev 2012 13 1183 1186 10.7314/APJCP.2012.13.4.1183 22799302

[B11] Park SW Hur SY Yoo NJ Lee SH Somatic frameshift mutations of bone morphogenic protein receptor 2 gene in gastric and colorectal cancers with microsatellite instability Apmis 2010 118 824 829 10.1111/j.1600-0463.2010.02670.x 20955454

[B12] Ye L Lewis-Russell JM Davies G Sanders AJ Kynaston H Jiang WG Hepatocyte growth factor up-regulates the expression of the bone morphogenetic protein (BMP) receptors, BMPR-IB and BMPR-II, in human prostate cancer cells Int J Oncol 2007 30 521 529 17203235

[B13] Pan L Baek S Edmonds PR Roach M 3rd Wolkov H Shah S Pollack A Hammond ME Dicker AP Vascular endothelial growth factor (VEGF) expression in locally advanced prostate cancer: secondary analysis of radiation therapy oncology group (RTOG) 8610 Radiat Oncol 2013 8 100 10.1186/1748-717X-8-100 23618468 PMC3653757

[B14] Zhang W Zhang M Zhou B Jia Z Qiao Z Zhang J Expression and significance of vascular endothelial growth factor C from multiple specimen sources in esophageal squamous cell carcinoma Int J Biol Markers 2012 27 e359 e365 10.5301/JBM.2012.9767 23125006

[B15] Sui X Kong N Ye L Han W Zhou J Zhang Q He C Pan H p38 and JNK MAPK pathways control the balance of apoptosis and autophagy in response to chemotherapeutic agents Cancer Lett 2014 344 174 179 10.1016/j.canlet.2013.11.019 24333738

[B16] O’Sullivan AW Wang JH Redmond HP P38 MAP kinase inhibition promotes primary tumour growth via VEGF independent mechanism World J Surg Oncol 2009 7 89 10.1186/1477-7819-7-89 19912664 PMC2785811

[B17] Roskoski R Jr Vascular endothelial growth factor (VEGF) signaling in tumor progression Crit Rev Oncol Hematol 2007 62 179 213 10.1016/j.critrevonc.2007.01.006 17324579

[B18] Raida M Clement JH Leek RD Ameri K Bicknell R Niederwieser D Harris AL Bone morphogenetic protein 2 (BMP-2) and induction of tumor angiogenesis J Cancer Res Clin Oncol 2005 131 741 750 10.1007/s00432-005-0024-1 16136355 PMC12161192

[B19] Wu JB Fu HQ Huang LZ Liu AW Zhang JX Effects of siRNA-targeting BMP-2 on the abilities of migration and invasion of human liver cancer SMMC7721 cellsand its mechanism Cancer Gene Ther 2011 18 20 25 10.1038/cgt.2010.55 20885449

